# Depicting the battle between nectarine and *Monilinia laxa*: the fruit developmental stage dictates the effectiveness of the host defenses and the pathogen’s infection strategies

**DOI:** 10.1038/s41438-020-00387-w

**Published:** 2020-10-01

**Authors:** Marta Balsells-Llauradó, Christian J. Silva, Josep Usall, Núria Vall-llaura, Sandra Serrano-Prieto, Neus Teixidó, Saskia D. Mesquida-Pesci, Antonieta de Cal, Barbara Blanco-Ulate, Rosario Torres

**Affiliations:** 1 IRTA, XaRTA-Postharvest, Edifici Fruitcentre, Parc Científic i Tecnològic Agroalimentari de Lleida, 25003 Lleida, Catalonia Spain; 2 Department of Plant Sciences, University of California, Davis, Davis, CA 95616 USA; 3 Department of Plant Protection, INIA, Ctra. de La Coruña Km. 7, 28040 Madrid, Community of Madrid Spain

**Keywords:** Transcriptomics, Biotic, Fungal genetics

## Abstract

Infections by the fungus *Monilinia laxa*, the main cause of brown rot in Europe, result in considerable losses of stone fruit. Herein, we present a comprehensive transcriptomic approach to unravel strategies deployed by nectarine fruit and *M. laxa* during their interaction. We used *M. laxa*-inoculated immature and mature fruit, which was resistant and susceptible to brown rot, respectively, to perform a dual RNA-Seq analysis. In immature fruit, host responses, pathogen biomass, and pathogen transcriptional activity peaked at 14–24 h post inoculation (hpi), at which point *M. laxa* appeared to switch its transcriptional response to either quiescence or death. Mature fruit experienced an exponential increase in host and pathogen activity beginning at 6 hpi. Functional analyses in both host and pathogen highlighted differences in stage-dependent strategies. For example, in immature fruit, *M. laxa* unsuccessfully employed carbohydrate-active enzymes (CAZymes) for penetration, which the fruit was able to combat with tightly regulated hormone responses and an oxidative burst that challenged the pathogen’s survival at later time points. In contrast, in mature fruit, *M. laxa* was more dependent on proteolytic effectors than CAZymes, and was able to invest in filamentous growth early during the interaction. Hormone analyses of mature fruit infected with *M. laxa* indicated that, while jasmonic acid activity was likely useful for defense, high ethylene activity may have promoted susceptibility through the induction of ripening processes. Lastly, we identified *M. laxa* genes that were highly induced in both quiescent and active infections and may serve as targets for control of brown rot.

## Introduction

*Monilinia laxa* is the main causal agent of brown rot in Europe, leading to important losses of stone fruit in the field and postharvest^1^. The worldwide yearly losses are estimated to be 1.7 M euros for peach and nectarine^2^ and 170 M USD for peach, cherry, and plum production^3^. The disease is controlled using several cultural practices (e.g., removing the overwintering inoculum), chemical fungicides in the orchard, treatments onto mummified fruit, and postharvest storage at low temperatures^1,4^. However, the gradual withdrawal of some fungicides driven by concerns about their negative impact on the environment and human health, the constant threat of the emergence of fungicide resistance, and the appearance of novel virulence alleles demonstrate the need for alternative methods for managing brown rot^4–6^. Prior to infection, *M. laxa* can remain latent or quiescent on flowers and fruit surfaces until favorable host factors (i.e., fruit developmental stage^7^), and environmental factors and other characteristics intrinsic to the stone fruit variety^8^, trigger the disease cycle^9^.

During fruit infection, *M. laxa* can overcome the need for wounds to infect and penetrate the plant cell. As a necrotrophic pathogen, *M. laxa* relies on the secretion of cell wall-degrading enzymes (CWDEs), such as pectin methyl esterases^10^, and possibly phytotoxins, although these compounds have not been fully identified yet^11^. After penetration, *M. laxa* colonizes the epidermis of the fruit with hyphae^12^ causing the collapse and disruption of cells, lysogenic cavities, and total degradation of the cuticle and epidermis, similar to the lesions caused by *M. fructicola*^13^.

Overall, fruit can be infected at any growth stage, but their susceptibility to brown rot increases with maturation, which results in a short postharvest life^14^. Hence, the activation of immune responses alongside the physicochemical properties of the fruit may determine the pathogen’s ability to infect and spread. Although these underlying mechanisms have not been fully elucidated, possible explanations could depend on changes in cell wall composition, volatiles, organic acids, and phenolic compounds^15,16^.

We hypothesize that *M. laxa* is able to adapt its infection strategies according to the nectarine developmental stage, resulting in either quiescent or disease progression, while the plant host can only establish effective defenses to restrict pathogen growth in fruit tissues that have not yet reached full maturity. Here, the fruit responses and pathogenicity mechanisms in the nectarine–*M. laxa* interaction were investigated as a function of the host developmental stage and time. Nectarine fruit was harvested at two different developmental stages (immature and mature) and inoculated with *M. laxa*. Disease development and ethylene production were assessed for 3 days. Thanks to the recent availability of the *M. laxa* 8L genome^17^, a comparative transcriptomics study was conducted on the nectarine–*M. laxa* pathosystem across four time points. This approach allowed us to identify not only host defense responses that were uniquely or highly induced in immature fruit during early infections, which may partially explain why these tissues are resistant to brown rot, but also key strategies employed by the fungus to either become established in tissues or colonize them, which may be targeted to control brown rot.

## Results

### Nectarine susceptibility to brown rot is developmentally controlled

**Fig. 1:**
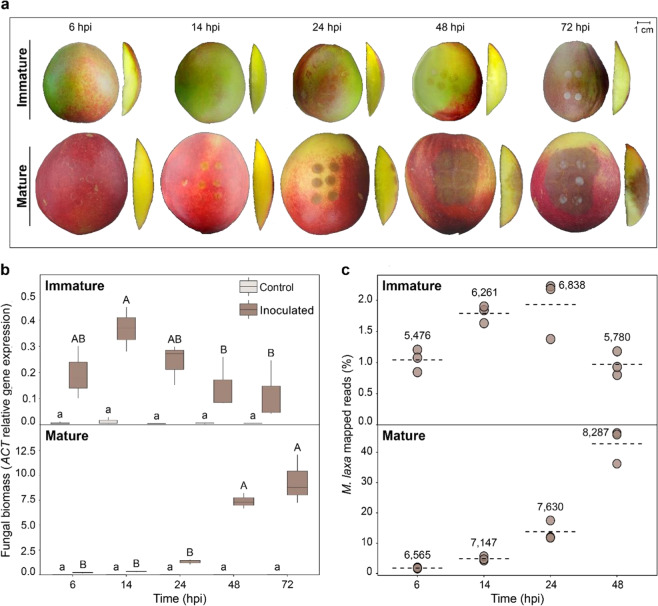
Fungal behavior development in “Venus” nectarines. **a** Brown rot spread development in immature and mature tissues at different time points after inoculation (6, 14, 24, 48, and 72 hpi). Two different viewpoints are shown (left image—entire fruit showing 6 drops; right image—perpendicular section of the fruit to discern fungus penetration if observable). **b** Determination of pathogen biomass by relative gene expression of the *M. laxa* reference gene (*ACT*), normalized to the expression of the nectarine reference gene (*TEF2*) in both stages (immature and mature) of both control (light brown) and inoculated (dark brown) tissues. The box plot represents the mean of three biological replicates with its interquartile range. Lowercase and uppercase letters indicate significance differences (*P* < 0.05, Student’s *T* test) in control and inoculated tissues, respectively. **c** Abundance (%) of *M. laxa* mapped reads in inoculated tissue out of the total amount of reads at each time point in both tissues. Each dot represents the number of mapped reads for each of the three biological replicates. The dashed line represents the average of the mapped reads in each group. Numbers represent the average of genes that were obtained at each time point in both tissues

We visually assessed the development of brown rot over time at two maturity stages of nectarine (Fig. 1a). Quality parameters were measured and summarized in Supplementary Table S1. Overall, the disease progressed in mature tissues, while only surface discoloration was observed in immature tissues. At the mature stage, tissue maceration was observed on the surface of the fruit at 14 hpi followed by the pathogen penetration of the pericarp tissues between 14 and 24 hpi, and increasing lesion spread at 48 and 72 hpi. Fungal biomass was also estimated in both inoculated and control (mock-inoculated) fruit to complement the visual assessments (Fig. 1b). Although no symptoms of brown rot disease were visible on the immature fruit surface at any time point, the *M. laxa* biomass increased from 6 to 14 hpi, when the highest quantity was detected, and then significantly decreased until 72 hpi. Although at early stages of infection (6–14 hpi), the fungal biomass was not significantly different between immature and mature tissues, it increased exponentially (*y* = 0.2119e^0.0596t^, *R*² = 0.9075) in the mature fruit at later time points, reaching levels approximately twenty times more than the maximum observed in immature fruit. In control tissues, a negligent quantity of the fungal biomass was detected across all time points in both stages.

A dual RNA-Seq study revealed the dynamics of the fruit–pathogen interaction at early (6 hpi and 14 hpi) and late (24 hpi and 48 hpi) infection time points. The expression of 21,334 nectarine genes (79.39% of total transcriptome) and 8364 *M. laxa* genes (87.30% of total transcriptome) was detected across all developmental stages and time points (Supplementary Table S2). The proportion of the total (i.e., from both host and pathogen) mapped reads for each sample that corresponded to *M. laxa* (Fig. 1c) strongly correlated (*r* = 0.996) with the measurements of fungal biomass. Remarkably, more than 6000 genes were found to be expressed in inoculated immature fruit at 14 hpi and 24 hpi, indicating that the pathogen was active in these tissues but yet it could not cause disease. More genes were detected in mature fruit, increasing across time, from 6565 at 6 hpi up to 8287 at 48 hpi, reflecting the progression of pathogen growth and host tissue colonization.

### Nectarine and *M. laxa* synchronize their transcriptional responses during their interaction

**Fig. 2:**
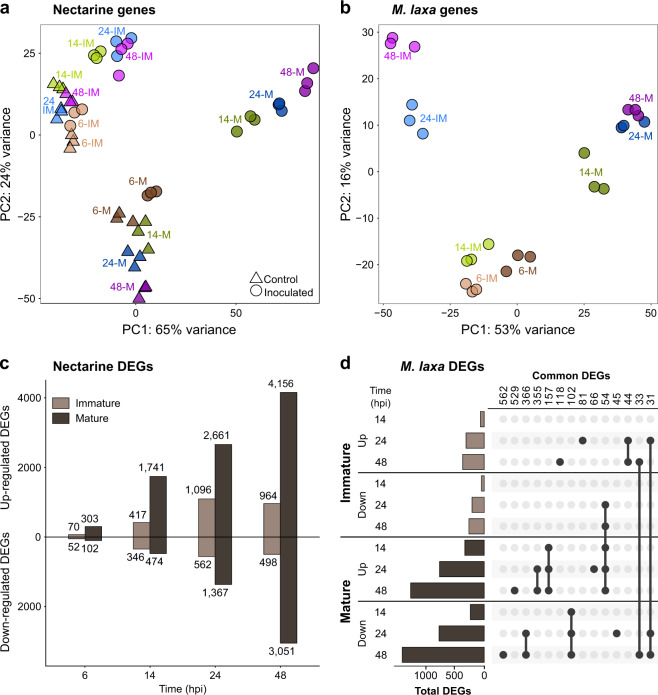
Nectarine and *M. laxa* gene expression profiles. **a**, **b** Patterns of gene expression represented by principal component analysis (PCA) plots of normalized count matrices for nectarine (**a**) and *M. laxa* (**b**), generated by DESeq2 through differential expression analysis for both control (△) and inoculated tissue (◯). Labels indicate the time point (6, 14, 24, and 48 hpi) in both immature (IM) and mature (M) stage. **c** Amount of nectarine differentially expressed genes (DEGs) as a result of the pairwise comparison of inoculated vs control tissue obtained in DESeq2 (*P-*adj value ≤0.05). The upper part shows the upregulated DEGs and the lower, the downregulated ones, of all the four time points analyzed for the immature (light brown) and the mature (dark brown). The number of DEGs in each set are shown. **d** Amount of *M. laxa* DEGs obtained through pairwise comparisons between 14, 24, and 48 hpi compared to 6 hpi in both immature (light brown) and mature (dark brown) tissue. The highest groups of DEGs number in each set are indicated. Dots and lines represent the common DEGs that were found between time points in each stage

The principal component analyses (PCA) revealed that in nectarine, PC1 and PC2 (89% cumulative variance) clearly separated the samples based on their developmental stage and infection status (Fig. 2a). Notably, at both development stages, 14 hpi was the time point when the inoculated samples appeared to experience a significant change in their expression profiles compared to the controls. These results demonstrate that early time points are critical for dictating the outcome of the interaction. For *M. laxa*, PC1 (53%) distinguished the samples based on the fruit developmental stage, while PC2 (16%) mainly divided the samples between early- and late-inoculation time points (Fig. 2b). In immature fruit, there was an evident switch in the pathogen’s transcriptional profile after 14 hpi, coinciding with the decrease in fungal biomass, and then continued to change up to 48 hpi. In mature fruit, *M. laxa* showed a change in gene expression between 6 and 14 hpi, when disease symptoms were first noticed on the fruit surface. Then, between 14 and 24 hpi, the pathogen altered its gene expression in mature fruit once again and retained most of these changes up to 48 hpi. Remarkably, the expression patterns of *M. laxa* at late time points of infection were highly divergent when infecting immature and mature tissues, suggesting that the pathogen utilizes different survival or infection mechanisms depending on the host developmental stage.

A differential gene expression (DE) analysis was performed to determine the responses of immature and mature fruit to *M. laxa*, and to identify specific strategies used by the pathogen at specific times of infection. Nectarine DE genes (DEGs) (*P*-adj ≤ 0.05) were identified in comparisons between inoculated and control fruit for each maturity stage and time point (Fig. 2c and Supplementary Table S3). A total of 4005 DEGs were detected in immature fruit across all time points, and of these the majority (63.60%) were upregulated in inoculated tissues. In immature fruit, the number of DEGs (up- and downregulated) progressively increased over time and peaked at 24 hpi; then, the changes in gene expression appeared to reach a slightly lower plateau at 48 hpi. Mature fruit displayed a stronger transcriptional response to *M. laxa* infection since a total of 13,855 DEGs (3.5-fold that from immature fruit) were detected at early and late time points. The number of DEGs in mature fruit continuously increased from 6 hpi to 48 hpi, indicating that the host tissues were undergoing a large transcriptional reprograming as the disease progressed.

*Monilinia laxa* DEGs were detected by comparing the expression profiles of the fungus at each time point against 6 hpi for immature and mature fruit, respectively (Fig. 2d and Supplementary Table S4). These comparisons allowed us to depict how the pathogen modified its transcriptional response based on the initial time point of the interaction when gene expression profiles of *M. laxa* were similar between immature and mature fruit (Fig. 2b). A total of 3160 DEGs (*P-*adj ≤ 0.05) were detected, with 895 DEGs identified in immature fruit and 2842 in mature fruit. A closer inspection of these DEGs corroborated the divergence observed in the PCA at later time points (Fig. 2d). For example, the largest group of *M. laxa* unique DEGs consisted of downregulated genes in mature tissue at 48 hpi, followed by the upregulated ones in the same conditions. The DE data were further validated by RT-qPCR using eight nectarine (*r* = 0.892, *P* = 2.2 × 10^−16^) and eight *M. laxa* (*r* = 0.915, *P* = 2.2 × 10^−16^) DEGs, as shown in Supplementary Table S5.

### Susceptible mature fruit display a stronger transcriptional response to *M. laxa* infection than resistant immature fruit

**Fig. 3:**
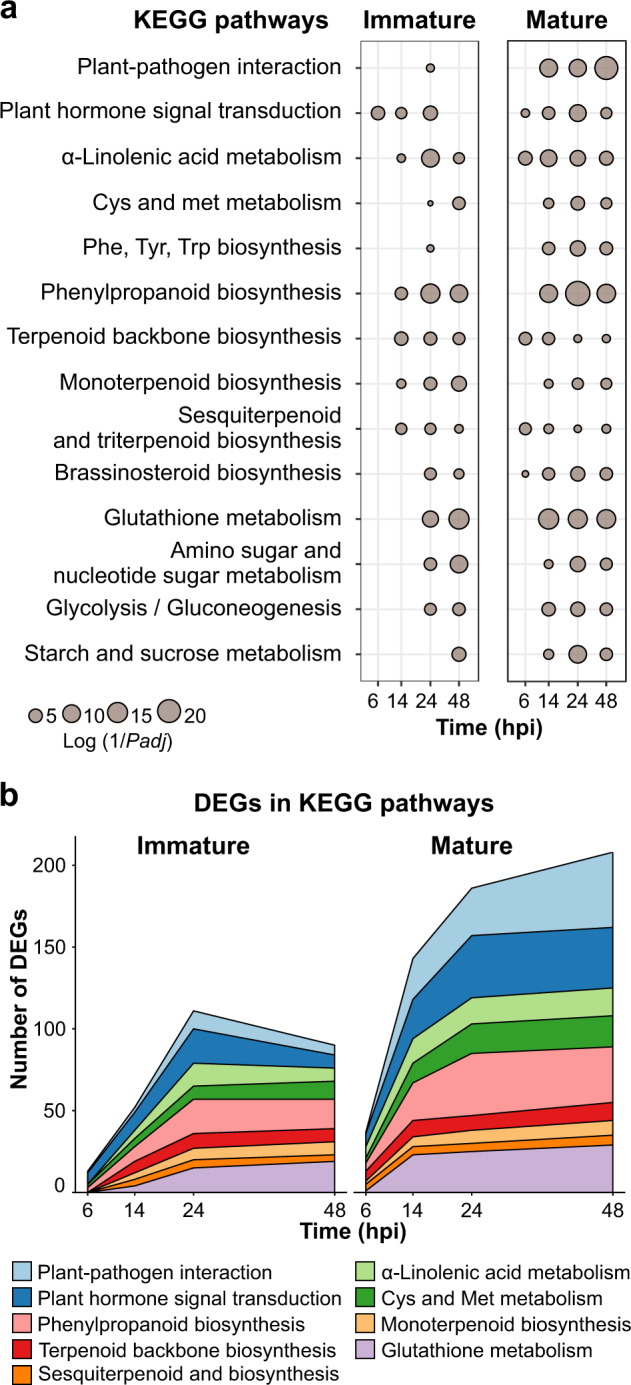
KEGG enrichments of upregulated genes in nectarine. **a** Metabolic pathways from the KEGG database that were found in at least half of the eight comparisons obtained in the differential expression analyses (inoculated vs control). The dot size represents the log of the inverted *P-*adj value obtained in the KEGG enrichment analyses along with all the time points in both stages (*P-*adj value ≤0.05) (Supplementary Table S3). **b** The magnitude of the fruit response in terms of the number of DEGs that have KEGG annotations for the selected metabolic pathways in both stages through time. Each color represents one different KEGG pathway

To study host metabolic pathways altered during *M. laxa* progression, we performed a functional enrichment analysis for KEGG terms in the upregulated nectarine DEGs at each time point for immature and mature fruit (Supplementary Table S3). Figure 3a depicts KEGG terms that were significantly enriched (*P-*adj ≤ 0.05) in at least four out of the eight comparisons (i.e., between mature and immature tissues and the four time points). In immature fruit, enriched pathways were more evident at or after 24 hpi. In contrast, multiple pathways were enriched in mature fruit, as shown by early time points, which suggests an overall activation of stress responses associated with the biotic challenge and tissue breakdown. These time-dependent responses to *M. laxa* were also evident when quantifying the number of DEGs for enriched categories related to plant defense (Fig. 3b), which confirmed that immature fruit had the highest gene expression induction at 24 hpi, and that mature fruit had a larger number of genes induced than immature fruit as early as 6 hpi. DEGs related to the plant–pathogen interaction pathway (e.g., *CERK1*, *PTI1*, *MAP2K1*, *WRKY33*) were largely absent from the immature fruit response, with the exception of 24 hpi, but were quite abundant in the mature fruit response starting at 14 hpi (Supplementary Table S3). Hormone signaling was enriched early in fruit at both developmental stages, though it appeared to become less relevant in immature fruit at 48 hpi. Cysteine and methionine metabolism and α-linolenic acid metabolism pathways, associated with ethylene (ET) biosynthesis and jasmonic acid (JA) biosynthesis, respectively, were enriched in both immature and mature fruit, though more prominently in the latter. Pathways related to the biosynthesis of terpenoids were also found to be enriched at early time points in immature (14 hpi) and mature fruit (6 hpi), but their enrichment was higher in immature than mature tissue. Other pathways that appeared to be relevant for nectarine responses against *M. laxa* included the phenylpropanoid and glutathione metabolism, which were highly induced in the mature fruit, likely utilized as antioxidants.

### Ethylene and jasmonic acid pathways are activated in response to *M. laxa* inoculations of nectarine

**Fig. 4:**
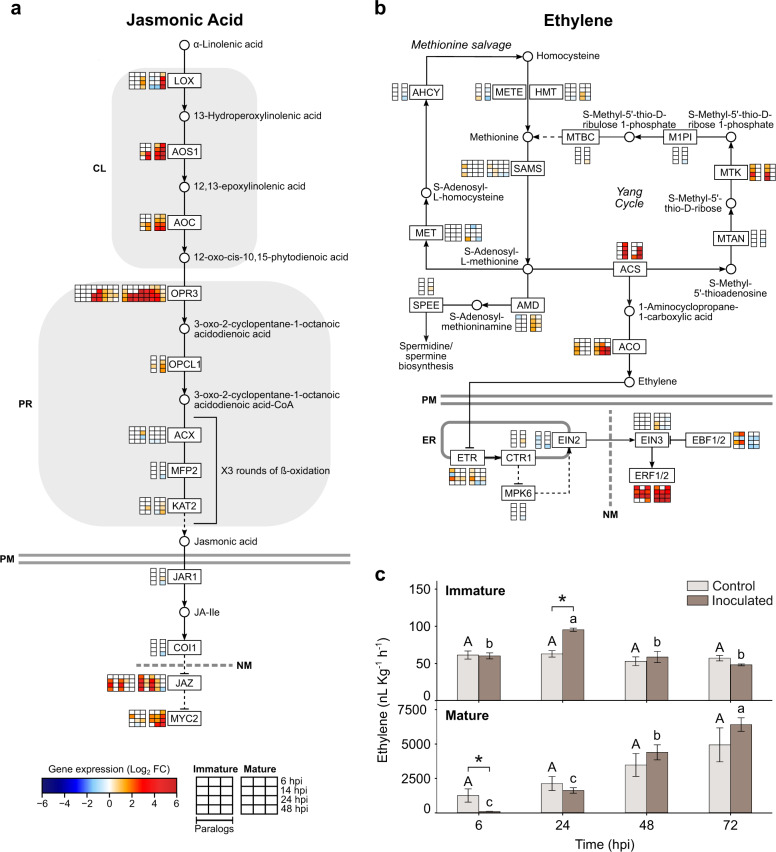
Activation of jasmonic and ethylene pathways in nectarine fruit after inoculations with *M.laxa*. **a**, **b** Jasmonic acid and ethylene pathways are shown with substrates (◯) and enzymes (boxes) and include 32 and 41 DEGs for JA and ET, respectively. The scale color of the heat maps represents the intensity of the significant expression changes (Log_2_FC), which resulted from the pairwise comparison of inoculated vs control samples (*P-*adj value ≤0.05). Paralogs of each analyzed enzyme are represented in columns and grouped by their expression in immature (left boxes) and mature (right boxes) at each time point (hpi). Dashed lines indicated that some steps had been omitted. PM plasmatic membrane, NM nucleus membrane, ER endoplasmic reticulum, CL chloroplast, PR peroxisome. Enzyme abbreviations and lists of paralogs genes for each protein are provided (Supplementary Table S3). **c** Ethylene measurements of the nectarine–*M. laxa* pathosystem through time. Values represent the mean (*n* = 4) and the vertical bars, the standard error. Symbols (*) indicate significant differences according to Student’s *T* test (*P* ≤ 0.05). Uppercase and lowercase letters indicate significant differences (*P* ≤ 0.05, Tukey’s test) in control and inoculated tissues, respectively

Given the enrichment of genes involved in plant hormone signaling transduction during early infection and the activation of methionine and α-linolenic metabolism in both fruit tissues across time, a targeted analysis of ET and JA pathways was conducted. The transcriptional activation of JA biosynthesis was evident in immature and mature fruit, with special emphasis in the induction of multiple genes encoding the initial biosynthetic steps (Fig. 4a), from lipoxygenase (*LOX*) to 12-oxophytodienoic acid reductase (*OPR3*). Later steps of the biosynthesis pathway were only moderately activated in both tissues. In mature tissues at 48 hpi, a downregulation of the JA-amino synthetase (*JAR1*) gene was observed, involved in the production of the active form of JA, and of the homolog of the JA receptor coronatine-insensitive protein 1 (*COI1*). Two out of the five paralogs of the signaling repressor JA ZIM domain (*JAZ*) appeared to be activated in immature and mature tissues at multiple time points. The three paralogs encoding the transcriptional activator of JA responses, *MYC2*, were strongly induced in mature fruit after 14 hpi and upregulated in immature fruit only at 14 hpi and 24 hpi. In fact, the *MYC2* gene expression level of the third paralog (*Prupe.5G130700.1*) was significantly higher in inoculated immature than mature tissue, but then, its expression was significantly higher in mature than immature tissue at both 24 and 48 hpi (Supplementary Table S3).

The steps committed to ET biosynthesis catalyzed by the 1-aminocyclopropane-1-carboxylate synthase (*ACS*) and the 1-aminocyclopropane- 1-carboxylate oxidase (*ACO*) genes were highly induced in response to *M. laxa* inoculations, particularly in mature fruit (Fig. 4b). The *ACS2* (*Prupe.5G106200.1*) and the *ACO3* (*Prupe.7G212000.1*) genes showed the highest upregulation (*ACS2* in both tissues and *ACO3* in mature tissue). Ethylene signal transduction elements (*ETR*, *CTR*, *EIN2*, and *EIN3*) showed only moderate changes in gene expression in response to the pathogen. Interestingly, although the negative regulator *EBF1/2* was downregulated at 14 and 48 hpi in both tissues, it was highly upregulated in immature tissue at 6 and 24 hpi. However, all three paralogs of the ET response factor 1/2 (*ERF1/2*), which control multiple ET responses and are a point of signal integration for JA and ET signal transduction, were highly upregulated in both tissues. The *ERF1/2* gene expression level of the second paralog (*Prupe.6G348700.1*) was significantly higher in mature inoculated than immature inoculated fruit at 14 hpi (data not shown).

In addition, the ET produced by *M. laxa*-inoculated and control fruit was measured to complement the transcriptional data (Fig. 4c). Control nectarines followed the ET pattern of a climacteric fruit; low and steady levels of ET in immature fruit and high and significantly increased levels in mature fruit until ripening. However, in inoculated immature fruit, ET production significantly peaked at 24 hpi, corresponding to the peak of transcriptional responses in this tissue, before returning to levels equivalent to the control fruit. In inoculated mature fruit, the ET production was significantly lower than control fruit at 6 hpi, but then significantly increased. These results suggest that nectarine was performing a tightly regulated response of ET.

### *Monilinia laxa* adapts its infection strategies according to the host environment conditions

**Fig. 5:**
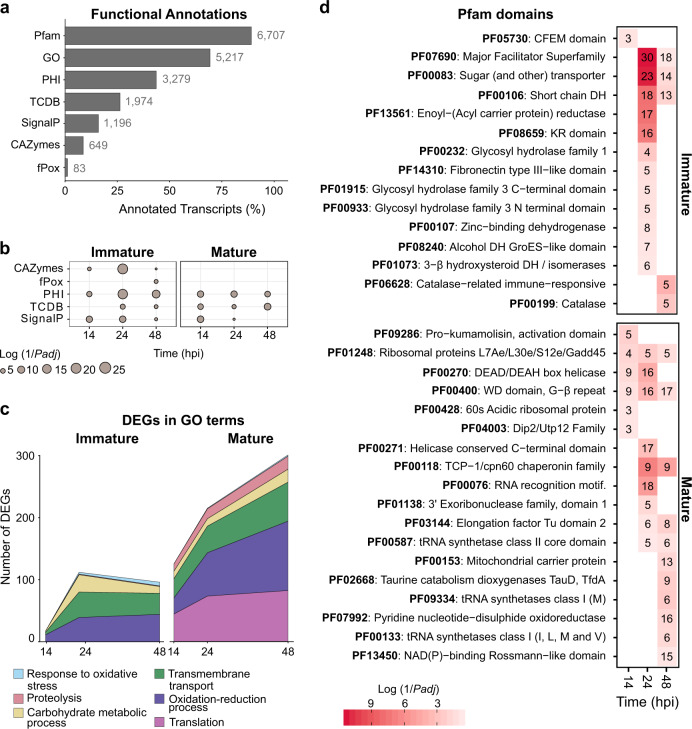
Summary of functional annotations and functional enrichments of *M.laxa*. **a** De novo functional annotations in all *M. laxa* transcripts obtained (9581) (Supplementary Table S4). Each category is represented by the proportion (%) of annotated transcripts across *M. laxa* transcriptome and the specific number of DEGs next to the bar. Pfam protein family database, GO gene ontology, PHI pathogen–host interaction, TCDB transporter classification database, SignalP presence of secretion signal peptides, CAZy carbohydrate-active enzyme, fPox fungal peroxidases. **b** Enrichment of functional categories across all time points in both tissues. Pairwise comparisons were performed between 14, 24, or 48 hpi compared to 6 hpi, for each maturation stage. The dot size represents their significance (log of the inverted *P*-adj value) obtained in Fisher tests. **c** The magnitude of *M. laxa* response in terms of number of DEGs (*P-*adj ≤ 0.05) that have GO terms for some relevant terms in both stages along time. Each color represents one different GO term. **d** Pfam enrichments of *M. laxa* genes that were overexpressed in 14, 24, and/or 48 hpi compared to 6 hpi, for each stage, obtained in DESeq2 (*P*-adj value ≤0.05) (Supplementary Table S4). The color scale of the heat maps represents the log of the inverted *P*-adj value. The number of DEGs in each Pfam are also shown

To determine which fungal genes and functions are biologically relevant during *M. laxa* interactions with nectarine, we performed a functional analysis of the pathogen transcriptome. First, a total of 9581 transcripts were de novo annotated for multiple functional categories, including carbohydrate-active enzymes (CAZymes), fungal peroxidases (fPox), genes involved in pathogen–host interactions (PHI), membrane transport proteins (TCBD), and proteins with signal peptides (SignalP), among others (Fig. 5a and Supplementary Table S4). Then, an enrichment analysis (Fisher, *P-*adj ≤0.05) of these large functional categories in the upregulated DEGs across infection was performed to obtain a general picture of specific gene categories induced by the pathogen in immature and mature fruit (Fig. 5b). In immature fruit, these large categories were enriched in *M. laxa* upregulated DEGs at least at one time point when compared to 6 hpi. Particularly at 24 hpi, a significant abundance of CAZymes and PHI genes was observed. Fungal peroxidases were only significantly enriched in immature fruit at 48 hpi. In contrast, enrichment of CAZymes and fungal peroxidases was not observed at any time point in mature tissues. Genes in involved in pathogen–host interactions and membrane transport remained enriched at relatively even levels from 14 to 48 hpi in mature fruit.

We identified GO terms related to pathogenicity, virulence, and fungal growth among the upregulated DEGs for each host developmental stage (Fig. 5c). Among this subset of biologically relevant GO terms, threefold more upregulated DEGs were detected when *M. laxa* was inoculated in mature fruit compared to immature fruit. Particularly, the number of *M. laxa* upregulated DEGs in immature tissue increased progressively until 24 hpi and then decreased slightly at 48 hpi, whereas in mature tissue, the upregulated DEGs increased along with infection time. Notably, these gene expression patterns resembled the transcriptional response of the host for each developmental stage (Fig. 3b). In both stages, *M. laxa* induced a high number of DEGs related to oxidative–reduction processes and transmembrane transport, although genes involved in protein translation and proteolysis were only abundantly expressed in mature tissue. However, genes involved in response to oxidative stress were mainly expressed in immature at 48 hpi, together with the enrichment of fungal peroxidases at this time point (Fig. 5b).

Lastly, the enrichments of Pfam domains (*P-*adj ≤ 0.05) were also carried out using the *M. laxa* upregulated DEGs (Fig. 5d and Supplementary Table S4). In agreement with previous results, Pfam categories were mainly enriched at 24 hpi in immature fruit, with the exception of proteins containing the fungal pathogenesis-related CFEM domain (PF05730), which were uniquely enriched earlier at 14 hpi. In addition, Pfam domains related to fungal membrane transport (PF07690 and PF00083) were largely prominent in immature fruit, especially at 24 hpi, where up to 53 genes were induced. Less significantly enriched, fungal glycosyl hydrolases, dehydrogenases (DH), and catalases were found at 48 hpi in immature tissues.

The number of enriched Pfam domains among *M. laxa* upregulated DEGs in mature fruit, such as those related to transcription and translation (e.g., PF03144 and PF00587), increased throughout disease progression (Fig. 5d). However, other relevant domains, such as some related to proteolysis activity (e.g., PF09286 Pro−kumamolisin domain), uniquely peaked at 14 hpi. Notably, upregulated DEGs annotated as ribosomal proteins and transcriptional factors (PF01248 and PF00400) involved in growth and cell cycle control were prevalent throughout infection of mature fruit. Later, infection time points exhibited enrichments of protein domains belonging to membrane transport (e.g., mitochondrial carrier protein) and redox functions (e.g., an oxidoreductase).

### Highly induced *M. laxa* genes during inoculation provide possible targets for disease control

To identify potential target genes for the control of *M. laxa*, a closer examination was conducted of the most highly *M. laxa* upregulated DEGs (i.e., largest Log_2_FC) from all time points and tissue comparisons (Table 1 and Supplementary Table S4). The top five *M. laxa*-induced DEGs in immature and mature fruit were unique between the tissue types, reinforcing the evidence that the pathogen displays a different behavior according to the developmental stage of the host. Strongly induced DEGs at 14 hpi unique to early infections of immature fruit included fungal phosphate transporters, phospholipases, and oxidoreductases. A member of the glycosidase hydrolase family 31 (*Monilinia_056600*) was highly expressed at 24 hpi in immature fruit, alongside a transmembrane fructose transporter (*Monilinia_074660*) and histidine phosphatase (*Monilinia_002270*). The highest induced DEGs in immature fruit were detected at 48 hpi and corresponded to an oxidoreductase gene (*Monilinia_010850*), a homolog of the alcohol oxidase (*OAX1*) from *Cladosporium fulvum*, and the same transmembrane fructose transporter (*Monilinia_074660*) already found at 24 hpi. Interestingly, *M. laxa* DEGs with fungal peroxidase annotations, a catalase (*Monilinia_039930*) and a haloperoxidase (*Monilinia_049900*), were only detected at 48 hpi in immature fruit.

**Table 1:** Top upregulated genes of *M. laxa*

Accession	Log_2_FC	Selected functional annotations
***Immature —14 HPI***		
*Monilinia_058830*	5.90	**TCDB**: 2.A.1.9.2 (Inorganic phosphate transporter) | **PHI**: *PHO84* (*Cryptococcus neoformans*, reduced virulence)
*Monilinia_028560*	5.37	**PFAM**: PF04185.14 (Phosphoesterase family) | **PHI**: *plcC* (*Mycobacterium tuberculosis*, unaffected pathogenicity) | **SignalP**: 0.811
*Monilinia_060140*	3.51	**SignalP**: 0.686
*Monilinia_079910*	3.12	**PFAM**: PF01633.20 (Choline/ethanolamine kinase)
*Monilinia_009770*	2.99	**PFAM**: PF00264.20 (Common central domain of tyrosinase) | **SignalP**: 0.667
***Immature —24 HPI***		
*Monilinia_056600*	8.45	**CAZy**: GH31 | **PHI**: *Gls2* (*Magnaporthe oryzae*, reduced virulence)
*Monilinia_074660*	8.09	**TCDB**: 2.A.1.1.69 (Sugar/H+ symporter) | **PHI**: *FRT1* (*Botrytis cinerea*, unaffected pathogenicity)
*Monilinia_002270*	7.72	**PFAM**: PF00300.22 (Histidine phosphatase superfamily (branch 1) | **PHI**: *FGSG_02549* (*Fusarium graminearum*, reduced virulence) | **SignalP**: 0.897
*Monilinia_033100*	7.68	**TCDB**: 2.A.1.1.119 (Putative uncharacterized protein *An14g04280*) | **PHI**: *MoST1* (*Magnaporthe oryzae*, unaffected pathogenicity)
*Monilinia_016250*	7.42	**TCDB**: 2.A.1.7.11 (Glucose/galactose transporter) | **PHI**: *PD0681* (*Xylella fastidiosa*, increased virulence) | **SignalP**: 0.632
***Immature—48 HPI***		
*Monilinia_010850*	9.53	**GO**: GO:0055114 (oxidation-reduction process) | **PFAM**: PF00732.19 (GMC oxidoreductase) **CAZy**: AA3-3 | **PHI**: *AOX1* (*Passalora fulva*, reduced virulence)
*Monilinia_074660*	8.77	**TCDB**: 2.A.1.1.69 (Sugar/H+ symporter) | **PHI**: *FRT1* (*Botrytis cinerea*, unaffected pathogenicity)
*Monilinia_022560*	7.92	**SignalP**: 0.844
*Monilinia_039930*	7.91	**fPox**: Catalase | **PHI**: *CAT1* (*Candida albicans*, reduced virulence)
*Monilinia_034450*	7.79	**CAZy:** GH3 | **PHI**: Avenacinase (*Gaeumannomyces graminis*, loss of pathogenicity) **SignalP**: 0.718
***Mature—14 HPI***		
*Monilinia_077490*	9.43	**GO**: GO:0006508 (proteolysis) | **PFAM**: PF01828.17 (Peptidase A4 family) | **SignalP**: 0.64
*Monilinia_037020*	6.77	**CAZy**: GH71 | **SignalP**: 0.886
*Monilinia_015240*	6.54	**TCDB**: 2.A.3.4.3 (GABA-specific permease) | **PHI**: *bcaP* (*Staphylococcus aureus*, unaffected pathogenicity / reduced virulence)
*Monilinia_000560*	6.10	**CAZy**: GH28 | **PHI**: *BcPG2* (*Botrytis cinerea*, reduced virulence) | **SignalP**: 0.837
*Monilinia_050850*	5.68	**GO**: GO:0006508 (proteolysis) | **PFAM**: PF09286.11 (Pro-kumamolisin, activation domain) **SignalP**: 0.84
***Mature—24 HPI***		
*Monilinia_077490*	8.90	**GO**: GO:0006508 (proteolysis) | **PFAM**: PF01828.17 (Peptidase A4 family) | **SignalP**: 0.64
*Monilinia_006190*	7.37	**PFAM**: PF00107.26 (Zinc-binding dehydrogenase)
*Monilinia_041700*	7.18	**CAZy**: GH28 | **PHI**: *PGX1* (*Cochliobolus carbonum*, unaffected pathogenicity) **SignalP**: 0.913
*Monilinia_041730*	6.89	**TCDB**: 2.A.1.14.38 (Uncharacterized transporter YIL166C) | **PHI**: *GzMyb019* (*Fusarium graminearum*, unaffected pathogenicity)
*Monilinia_073540*	6.74	**CAZy**: AA7 | **PHI**: *ZEB1* (*Fusarium graminearum*, unaffected pathogenicity) | **SignalP**: 0.778
***Mature—48 HPI***		
*Monilinia_077490*	9.25	**GO**: GO:0006508 (proteolysis) | **PFAM**: PF01828.17 (Peptidase A4 family) | **SignalP**: 0.64
*Monilinia_041700*	7.99	**CAZy**: GH28 | **PHI**: *PGX1* (*Cochliobolus carbonum*, unaffected pathogenicity) **SignalP**: 0.913
*Monilinia_068440*	7.64	None
*Monilinia_013220*	7.46	**GO**: GO:0055114 (oxidation-reduction process) | **PFAM**: PF00743.19 (Flavin-binding monooxygenase-like) | **PHI**: *iaaM* (*Pseudomonas savastanoi*, reduced virulence)
*Monilinia_041730*	7.42	**TCDB**: 2.A.1.14.38 (Uncharacterized transporter YIL166C) | **PHI**: *GzMyb019* (*Fusarium graminearum*, unaffected pathogenicity)

Represented genes are the five most upregulated genes, obtained in the pairwise comparisons generated by DESeq2. Values correspond to the expression (log_2_FC) of each time point (14, 24, and 48 hpi) compared to 6 hpi of both immature and mature fruit. The accession number of genes and selected functional annotations for each gene are also shown. *TCDB* Transporter Classification Database; *PHI* Pathogen-Host Interaction; *Pfam* Protein Family database; *SignalP* Presence of secretion signal peptides; *CAZy* Carbohydrate-Active enzyme; *GO* Gene Ontology; *fPox* fungal peroxidases

In mature fruit, a single protease gene (*Monilinia_077490*) was the highest upregulated *M. laxa* DEG at all time points. Two polygalacturonases (glycoside hydrolase family 28) were among the largest induced DEGs during infections of mature fruit; *Monilinia_000560* was highly upregulated at 14 hpi, whereas *Monilinia_041700* was highly expressed at 24 and 48 hpi. Another CAZyme (glycoside hydrolase family 71, *Monilinia_037020*) was also highly enriched at 14 and 24 hpi. In mature tissue, transporters and hormone-related genes were among the highest expressed DEGs. An amino acid transporter (*Monilinia_015240*) was significantly expressed at 14 hpi, while a tryptophan 2-monooxygenase (*Monilinia_013220*) was induced at 48 hpi, known to be involved in virulence in another pathosystem^18^. Altogether, these results suggest that targeting of specific genes involved in response to oxidative stress, nutrient transport, and carbohydrate catabolism may reduce quiescent infections, while specific proteolytic genes and additional CAZymes may help inhibit or reduce the severity of disease in susceptible fruit.

## Discussion

The first line of plant defense that *M. laxa* has to overcome is the constitutive physical (e.g., cuticle and plant cell wall) and chemical barriers (e.g., preformed antifungal compounds) present in the fruit surface. The developmental process from immature to mature fruit is characterized by physical and chemical changes in fruit firmness, leading to softening at the onset of ripening^19^. In fact, the flesh firmness of immature fruit was higher than the mature fruit (Supplementary Table S1). *Monilinia laxa* appeared to produce more CWDE (e.g., CAZymes) in immature fruit, which suggests that the pathogen could be trying harder to overcome the host cell walls in these tissues. Nevertheless, the immature tissue had no visible disease symptoms. Other alterations occurring during fruit development include changes in plant cuticle, sugar accumulation, volatile compounds, and secondary metabolites synthesis, which have been reviewed as promoting susceptibility to pathogens in ripening fruit. Hence, higher soluble solids content and lower titratable acidity on mature fruit (Supplementary Table S1) could favor pathogen colonization.

Plant–pathogen interactions take place when pathogen-associated molecular patterns (PAMP) are recognized by the plant’s pattern recognition receptors^20^, which ultimately triggers a defense response known as PAMP-triggered immunity (PTI)^21^. The chitin elicitor receptor kinase 1 (*CERK1*)^22^ (*Prupe.3G213100.1*) was upregulated in the mature tissue at 14 hpi. Also, the expression levels of the transcriptional activator *PTI5* (*Prupe.4G055500.1*) were up to 2.5-fold and 5-fold higher in mature fruit when compared to immature fruit, at 24 and 48 hpi, respectively. PTI responses can be suppressed by effector proteins secreted by the pathogen, which in turn, will elicit effector-triggered immunity (ETI)^23^. In our pathosystem, proteins with the CFEM domain (Pfam PF05730) and signal peptides were enriched in the early infection stage (14 hpi) on immature tissue. Among the annotated genes with the CFEM domain, the *Monilinia_077410* is a homolog of *BcCFEM1* from *B. cinerea*, an effector shared by many *Botrytis* spp.^24^ and described to be important for its virulence^25^. These results suggest that *M. laxa* may secrete some type of effector proteins in immature fruit.

Once the host–pathogen interaction began, both pathogen and host triggered their own transcriptional reprograming. In mature tissue, both nectarine and *M. laxa* abruptly changed their gene expression profile at 14 hpi, coinciding with the ability of the pathogen to grow and macerate the fruit tissues within 14 h. From 14 hpi onwards, the pathogen started to penetrate and switched toward an aggressive necrotrophic phase, which was retained at later infection times. Functions related to transmembrane transport, oxidation-reduction process, and translation were among the most abundant activities in mature fruit, denoting the growth and spread of the pathogen. In contrast, the number of nectarine and *M. laxa* DEGs in immature fruit remained somewhat steady through infection time, even when fungal biomass peaked at 24 hpi. Overall, these findings suggest that inoculated mature nectarines displayed an earlier and broader response to *M. laxa* than immature ones, likely due to the faster pathogen growth and virulence mechanisms activation in these tissues.

Both PTI and ETI are able to induce the host hormone signaling transduction pathway^21^, which was found to be enriched, starting at 6 hpi in both tissues. Jasmonic acid and ET are known to be involved in defense responses against necrotrophs, such as mediating the host’s responses against them^26^, but ET is also required for fruit ripening and senescence processes, which are conducive to disease susceptibility^21,27,28^. Jasmonic acid can also mediate the disease resistance of fruit by increasing the fruit antioxidant capacity^29^, but some fungi are able to hijack the JA signaling pathway to cause disease^30^. Although the early steps of JA biosynthesis were highly induced upon *M. laxa* inoculation, downregulation of receptor genes was observed in mature fruit inoculated with *M. laxa* when compared to controls. These findings suggest that *M. laxa* could be somehow blocking the JA signaling pathway, although the mechanisms involved are unknown.

Ethylene biosynthesis increases during ripening of climacteric fruit^31^, such as nectarines. In our study, the control immature fruit (system 1, associated with fruit development) produced basal ethylene levels, whereas ethylene production in control mature fruit (system 2, involved in ripening) increased through time after harvest. In inoculated immature fruit, there was a significant peak of ET production as compared to the control at 24 hpi. This discrete induction of ET can be part of the fruit defense responses against *M. laxa*. Alternatively, the pathogen could be inducing fruit ethylene biosynthesis in immature fruit to accelerate ripening, in an attempt to promote fruit physicochemical changes that are conducive to disease^28^. Along this line, *ACS2* and *ACO1*, involved in system 2 ET production^32^, were overexpressed in inoculated immature tissues. Previous studies have reported on a similar modulation of ET biosynthesis by the pathogen^33^. However, after 24 hpi, ethylene levels in inoculated immature fruit fell to control levels, and the fruit remained resistant. This may be in part due to the upregulation of the ethylene signaling inhibitors *EBF1/2*, which could mitigate the ethylene-induced ripening processes that contribute to susceptibility. In contrast, in inoculated mature fruit, ET production and signal transduction were lower at 6 hpi in inoculated fruit but grater from 24 hpi onward, following the autocatalytic system 2 ethylene biosynthesis. Overall, the results indicate the ability of *M. laxa* to differentially alter ET production to promote susceptibility and, in turn, the ability for immature fruit, but not mature fruit, to mitigate the consequences of this induction^27^.

The above observations indicate that although *M. laxa* was deploying some strategies to infect the immature tissues, it was not able to overcome either the surface or the active defense responses deployed by the immature fruit. *Monilinia laxa* remained on the immature tissue, increasing its biomass and multiplying on the surface, until 14 hpi when it ceased to grow. It is known that *Monilinia* spp. can remain quiescent on fruit surfaces^9^ and that they can employ appressoria as resting structures on immature nectarines^12^. After 14 hpi, *M. laxa* biomass and reads started to decrease, switching its transcriptional machinery by employing different sets of genes in order to deploy different strategies to survive on the fruit’s surface. Some results point out that *M. laxa* could either be starting a quiescence period or moving toward an autolysis process, breaking cells to feed on its remains. Another possibility is that the remaining *M. laxa* cells on immature fruit were being attacked by the host defenses. This is supported by the expression of *M. laxa* genes associated with response to oxidative stress at late time points, such as catalases, previously reported in detoxification during infection of tomato leaves by *B. cinerea*^34^. Thus, it is likely that immature fruit was generating reactive oxygen species (ROS) during the interaction through an oxidative burst^35^ to kill the pathogen.

*Monilinia laxa* could also be producing ROS for its development and as a pathogenicity mechanism to damage the host tissue. Particularly, the NADPH oxidase (Nox) complex is involved in both fungal ROS production and its use in sclerotia development and virulence^36,37^. Some genes encoding the Nox regulator R (NoxR) (e.g., *Monilinia_061250* and *Monilinia_079620*) were found to be upregulated at 24 hpi in both mature and immature tissue. At later stages, a highly induced alcohol oxidase expressed in immature tissue at 48 hpi could be another ROS producer, previously described as an alternative ROS production system. Lin et al.^38^ demonstrated that *AOX1* was involved in pathogenicity and oxygen stress responses in *B. cinerea*. Concomitantly, nectarine counteracted the pathogen oxidative burst by expressing genes of antioxidant metabolism compounds such as glutathione and redox-related amino acids (Cys and Met).

Plant secondary metabolites such as terpenoids have been described to protect the fruit under biotic and abiotic stresses^39^, although their role can be tissue-dependent. Overall, the enrichment of genes involved in secondary metabolite biosynthesis was higher in resistant immature than susceptible mature tissue, which suggests that either the host was producing terpenoids in the resistant immature tissue to prevent the attack or that *M. laxa* was inhibiting its biosynthesis on mature tissue. *Monilinia laxa* could also be able to degrade and transform terpenoids as described for *B. cinerea*^40^. The phenylpropanoid metabolism is also triggered in response to brown rot. In both immature and mature fruit, from 14 hpi to 48 hpi, phenylpropanoid-related pathways were highly induced. While on the immature tissue, these pathways could be involved in reinforcing the cell wall through lignin production^41^, the role in the mature fruit could be more focused on the detoxification of fungal ROS production^39^. Nevertheless, these hypotheses need to be further tested.

On mature nectarines, *M. laxa* deployed other virulence factors in addition to ROS production and scavenging. The pathogen expressed upregulated DEGs related to proteolytic activity, containing domains such as the Pro−kumamolisin domain (PF09286). The list of genes summarized in Table 1 could be putative pathogen target genes as they were expressed only when *M. laxa* infected the mature tissues, as none of the top five upregulated genes in mature tissue was found in the immature fruit. For instance, the highest expressed protease (*Monilinia_077490*) at in all time points is a homolog of a non-aspartyl protease (*ACP1*) found during pathogenesis in *Sclerotinia sclerotiorum*^42^. Cell wall-degrading enzymes are commonly produced by necrotrophic fungi as virulence factors and their secretion by *Monilinia* spp. on culture media has been previously reported^11^. A rhamnogalacturonan hydrolase (glycoside hydrolase family 28, *Monilinia_041700*), which was highly expressed at both 24 and 48 hpi, was already described as a putative virulence factor in *M. laxa* infecting peaches^43^.

Current information regarding the strategies utilized by either *Monilinia* spp. or stone fruit or during their interaction is mainly focused on specific metabolic pathways or actions developed by one of the two players. As a novel feature of the present research, we demonstrated the synchronized responses from nectarine and *M. laxa*, by utilizing a resistant immature and susceptible mature fruit throughout a course of infection. Future research studies should be focused on delving into the host defense system for the ongoing development of nectarine cultivars with increased resistance to brown rot, as well as conducting in-depth fungal studies to alter the ability of *M. laxa* to cause disease.

## Materials and methods

### Plant material and fungal culture

“Venus” nectarines (*P. persica* var. *nucipersica* (Borkh.) Schneider) were obtained from an organic orchard located in Raïmat (Lleida, Spain). Fruit was bagged 6 weeks before the last harvest and then harvested at two different fruit developmental stages, “mature” (211 Julian days) and “immature” (184 Julian days), and used immediately after harvest. Injured or deformed fruit was discarded, and fruit for analysis was further homogenized by using a portable DA-Meter (TR-Turoni, Forli, Italy), based on the single index of absorbance difference (*I*_AD_ = 1.99–2.26 for immature fruit and *I*_AD_ = 0.25–1.60 for mature fruit). Other assessments of quality parameters were performed on 20 randomly selected fruit (weight, cheek diameter, flesh firmness, soluble solids content, and titratable acidity), according to the method of Baró-Montel et al.^44^.

The *M. laxa* single-spore strain 8L (ML8L, Spanish Culture Type Collection number CECT 21100) was used for all experiments. Fungal conidial suspensions were maintained and prepared, as described by Baró-Montel et al.^43^.

### Fruit inoculations

Each fruit was inoculated with the application of six 30-μL drops of a conidial suspension at a concentration of 10^6^ conidia mL^−1^ on the fruit surface. Mock-inoculated fruits were equally treated with sterile water containing 0.01% (w/v) Tween-80. Fruit were placed in closed containers with a relative humidity of 97 ± 3% at 20 ± 1 °C. Four replicates consisting of five fruit per treatment were obtained at each sampling point (6, 14, 24, 48, and 72 hpi). Six cylinders of peel and pulp tissue (1-cm diameter and depth) encompassing the inoculation sites were sampled from each fruit and pooled for each replicate. Samples were immediately flash-frozen in liquid nitrogen and stored at −80 °C until extraction. For symptom analysis, inoculated fruit was imaged at the set time points. Ethylene production of both mock and *M. laxa* inoculated immature and mature fruit was determined, as described by Baró-Montel^33^.

### Fruit and fungal RNA extraction

Frozen samples were ground using a mortar and pestle. The total RNA was extracted following the protocol described previously^43^. Contaminant DNA was removed by treating RNA extracts with Turbo DNA-free DNase (Ambion, TX, USA). RNA concentration and purity were assessed with the Qubit^®^ 3.0 Fluorometer (Invitrogen, USA). Gel electrophoresis on an agarose gel stained with GelRed™ Nucleic Acid Gel Stain (Biotium, Hayward, CA, USA) was used to confirm the RNA was free of DNA and not degraded.

### cDNA libraries preparation and RNA sequencing

A total of 48 samples were analyzed by RNA sequencing, using three replicates of each treatment and stage at four of the sampled time points (6, 14, 24, and 48 hpi). cDNA libraries were prepared using the Illumina TruSeq RNA Sample Preparation Kit v2 (Illumina, USA). Quality control of the cDNA libraries was performed with the High Sensitivity DNA Analysis Kit in the Agilent 2100 Bioanalyzer (Agilent Technologies, USA). Paired-end libraries of 150-bp were sequenced on the Illumina HiSeq 4000 platform in IDSEQ INC (Davis, CA, USA).

### RNA-Seq bioinformatics pipeline and data processing

Quality and adapter trimming on raw reads were performed with Trimmomatic v0.33 (ref. ^45^) with the following parameters: maximum seed mismatches = 2, palindrome clip threshold = 30, simple clip threshold = 10, minimum leading quality = 3, minimum trailing quality = 3, window size = 4, required quality = 15, and minimum length = 36. Basic quality measurements were assessed with FastQC (https://www.bioinformatics.babraham.ac.uk/projects/fastqc/) before and after quality trimming. Mapping of parsed reads to a combined transcriptome of nectarine and *M. laxa* was performed using Bowtie2 (ref. ^46^). The nectarine transcriptome was obtained for peach (*Prunus persica* v2.0.a1) from the Genome Database for Rosaceae^47,48^ (https://www.rosaceae.org/species/prunus_persica/genome_v2.0.a1) as no nectarine genome was available. The transcriptome of *M. laxa* was previously obtained by our group^17^.

Count matrices were made from the Bowtie2 results using sam2counts.py v0.919 (https://github.com/vsbuffalo/sam2counts) and are available in Supplementary Tables S6 and S7 for nectarine and *M. laxa*, respectively. Differential expression (DE) analyses were conducted with the Bioconductor package DESeq2 (ref. ^49^) in R. Reads were first normalized for library size. Differentially expressed genes (DEGs) were considered to be those with an adjusted *P-*value less than or equal to 0.05. Two principal component analyses (PCA) were constructed with DESeq2 using the “plotPCA” function after normalized data sets were transformed with the “vst” function separately for nectarine and *M. laxa*.

### Functional analysis of nectarine genes

Functional annotations for the nectarine transcriptome were downloaded and processed from the Genome Database for Rosaceae version Peach v2.0.a1 (v2.1)^47,48^. Once differential expression analysis was combined with the functional annotations, enrichment analysis of KEGG (Kyoto Encyclopedia of Genes and Genomes) pathways was performed using Fisher’s exact test (*P* ≤ 0.05).

### Functional annotation and analysis of *M. laxa* genes

Transcripts were annotated with multiple databases. Gene ontology (GO) terms were obtained via Blast2GO (https://www.blast2go.com/). Additional BLAST searches were carried out to the transporter classification database (TCDB, http://www.tcdb.org/) and the pathogen–host interactions database (PHI, http://www.phi-base.org/). Custom HMMER alignment results for HMM profiles from the protein families database (Pfam), the carbohydrate-active enzyme annotation database (dbCAN, http://csbl.bmb.uga.edu/dbCAN/), and the fungal peroxidases database (fPox, http://peroxidase.riceblast.snu.ac.kr/) were similarly included. The presence of secretion signal peptides was evaluated for all genes in the transcriptome using SignalP v.4.0 (ref. ^50^). An *e*-value of 1e-3 was used as the cutoff value across all methods described. All enrichments carried out for *M. laxa* were performed as previously described for nectarine.

### Gene expression analysis with RT-qPCR and primer design

To determinate the fungal biomass in all samples and to validate RNA-Seq results, gene expression analyses with RT-qPCR were carried out. First-strand cDNA was synthesized on 1 µg of RNA using the M-MLV Reverse Transcriptase (Promega, USA) in the SimpliAmp Thermal Cycler (Applied Biosystems, USA). Expression of the reference genes was quantified through real-time quantitative PCR (RT-qPCR) using KAPA SYBR^®^ Fast qPCR Master Mix (Kapa Biosystems, Inc., Wilmington, USA) in the 7500 Real Time PCR System (Applied Biosystems, USA) with 2 µL of cDNA. Relative expression levels for fungal biomass determination were calculated according to the relative gene expression of the *M. laxa* reference gene *ACT* normalized to the nectarine reference gene expression *TEF2*. Primers for genes of interest were obtained from literature or designed *de novo* and are available in Supplementary Table S8. Primer efficiency was determined by the serial dilution method, using a mix of all cDNA samples as a template.

## Supplementary information

Suppl. Table S1

Suppl. Table S2

Suppl. Table S3

Suppl. Table S4

Suppl. Table S5

Suppl. Table S6

Suppl. Table S7

Suppl. Table S8

## Data Availability

The raw sequencing reads and the read mapping count matrices have been deposited in the National Center for Biotechnology Information Gene Expression Omnibus database under the accession GSE146293.
