# Supplementary material for: Depicting the battle between nectarine and Monilinia laxa: the fruit developmental stage dictates the effectiveness of the host defenses and the pathogen’s infection strategies
Source: Hortic Res. 2020 Oct 1;7:167. doi: 10.1038/s41438-020-00387-w (PMC7527454; doi:10.1038/s41438-020-00387-w)
Supplement: Supplementary file 1 — Suppl. Table S1 [file 41438_2020_387_MOESM1_ESM.docx]

**Supplementary Table S1.**  Summary of fruit quality parameters: harvest date, minimum and maximum values of single index of absorbance difference (I_AD_), weight, cheek diameter (CD), flesh firmness (FF), soluble solids content (SSC) and titratable acidity (TA) of ‘Venus’ nectarine cultivar. Data represent the mean (n = 20) ± the Standard Error.

| Stage | Harvest date^a^  (Julian days) | I_AD_ | Weight  (g) | CD  (mm) | FF  (N) | SSC  (° Brix) | TA  (g malic acid L^-1^) |
| --- | --- | --- | --- | --- | --- | --- | --- |
|  |  |  |  |  |  |  |  |
| Immature | 184 | 1.99 – 2.26 | 70.98 ± 2.56 | 48.95 ± 0.59 | 108.76 ± 1.91 | 9.83 ± 0.19 | 4.43 ± 0.07 |
| Mature | 211 | 0.25 – 1.60 | 187.65 ± 10.60 | 67.71 ± 1.30 | 74.43 ± 2.74 | 13.34 ± 0.32 | 2.87 ± 0.05 |
|  |  |  |  |  |  |  |  |

Maturity date is expressed as Julian days (e.g., January 1^st^ is considered as day 1).
